# Heterogeneous areas—identification of outliers and calculation of soil sampling uncertainty using the modified RANOVA method

**DOI:** 10.1007/s10661-016-5584-9

**Published:** 2016-09-22

**Authors:** Sabina Dołęgowska, Agnieszka Gałuszka, Zdzisław M. Migaszewski

**Affiliations:** Geochemistry and the Environment Division, Institute of Chemistry, Jan Kochanowski University, 25-406 Kielce, Poland

**Keywords:** Uncertainty, Outliers, Modified RANOVA, Soil samples

## Abstract

We modified the robust analysis of variance (RANOVA) method to calculate sampling uncertainty of selected trace elements determined in soil samples from two heterogeneous remote historic metal ore mining areas. Classical RANOVA is down-weighting the outlying values by replacing them during the calculation process with mean *± c·σ*_*r*_. Because the arithmetic mean is greatly influenced by outliers, it cannot represent a robust statistic. The main novel contribution of this work is use of median value that is independent on outliers and replace all extreme values during the calculation process with median *±* 2·*σ*_*r*_. In our work, 18 duplicate, composite soil samples were collected, digested with aqua regia in a closed microwave system, and analyzed twice for selected trace elements. To extract homogenous groups within sampling areas and make the results more accessible for interpretation, a cluster analysis was done. Subsequently, histograms of each element were prepared and statistical tests were applied to determine the normal distribution of datasets. For abnormally distributed elements, the outlying values were identified by four different methods: boxplot, mean *± c·σ*_*r*_, mean *± c·σ*, and median *±* 2·*σ*_*r*_. For five elements, the amount of outliers identified by the median ± 2·*σ*_*r*_ procedure was less than 10 %, and for these elements, the sampling uncertainty was computed using a modified RANOVA method. The sampling uncertainty computed with this method was 28.9 % for Cd, 15.2 % for Co, 14.5 % for Mn, 12.7 % for Ni, and 16.3 % for Zn, whereas that computed with a traditional model was 16.7 % for Cd, 9.2 % for Co, 20.5 % for Mn, 17.9 % for Ni, and 16.3 % for Zn.

**Electronic supplementary material:**

The online version of this article (doi:10.1007/s10661-016-5584-9) contains supplementary material, which is available to authorized users.

## Introduction

In environmental studies, the main errors are usually connected with the sampling step (Ramsey and Argyraki 1997; Petersen et al. 2005; Ramsey and Ellison 2007). The environmental parameters examined, including element concentrations, may vary with time, sampling season, temperature, and geology and topography of sampling sites. In general, these types of errors are usually very difficult to predict and can be extremely large, so the estimation of sampling uncertainty, next to analytical uncertainty, has become a standard procedure in each analytical method (Grøn et al. 2007; Lyn et al. 2007a, b; Joint Committee…. 2008; Reiter et al. 2011; Buczko et al. 2012; Esbensen and Wagner 2014). Routine and complex calculation of individual components of the total uncertainty includes the following steps: (i) testing of data distribution, (ii) identification of outliers in datasets showing abnormal distribution, (iii) data transformation (when the amount of outliers in dataset exceeds 10 % of total results), and (iv) calculation of uncertainty with a classical or robust analysis of variance (Dołęgowska et al. 2015).

The results derived from analysis of environmental samples are scarcely normally distributed. Because the normal distribution plays a significant role in statistical assessment of uncertainty, the first step relating to analysis of data distribution and identification of extreme values is crucial (Kuikin 2003; Reimann et al. 2005; Filzmoser et al. 2008). The presence of more than 10 % of outliers in dataset forces some additional mathematical operations that have to be done before using a classical analysis of variance. Data need to be transformed to obtain better symmetry and achieve normality (Reimann and Filzmoser 2000; Lee and Ramsey 2001; Filzmoser et al. 2009a; Dołęgowska et al. 2015), because environmental data are commonly positively skewed so the square root or logarithm transformation is typical in a conversion process. In practice, the log transformation is mainly used for positively skewed data, whereas the square root for slightly positively skewed data (Reiter et al. 2011), but as shown by Reimann and Filzmoser (2000), both these operations may fail to restore normality. They may reduce skewness but cannot accommodate the compositional nature of the data (Reimann et al. 2008; Filzmoser et al. 2009b; Filzmoser et al. 2012). It should be stressed that an effective transformation should give the closest to normally distributed dataset and depends on the type of distribution shape. When the amount of extreme values does not exceed 10 % of the total results, the sampling uncertainty can be calculated using a robust analysis of variance, which is less susceptible to extreme values. At this stage, the main problem is associated with correct identification of extreme values (Reimann and Garrett 2005; Rostron and Ramsey 2012).

Considering this, the estimation of sampling uncertainty is easy when the following prerequisites are met: (i) data are normally distributed, (ii) abnormality may be eliminated employing common transformation methods (e.g., log transformation), (iii) the amount of outliers (<10 % of total results) allows us to use the robust analysis of variance, and (iv) the presence of outliers does not result from specificity of sampling area. When these assumptions are not fulfilled, the calculation process is more complicated. The principal objectives of this study were to (i) assess the heterogeneity of sampling area using a cluster analysis method, (ii) identify the amount of outliers by four different methods, and (iii) estimate the level of sampling uncertainty for selected elements determined in soil samples collected within heterogeneous remote historic metal ore mining areas using a modified robust analysis of variance (RANOVA) method.

### When the RANOVA method can be applied?

Interpretation of results from analysis of environmental samples is a process that needs a multifaceted approach (de Zorzi et al. 2002; Barbizzi et al. 2004; Reimann et al. 2005; Buczko et al. 2012; Dołęgowska and Migaszewski 2013; Gałuszka et al. 2015). Before computing the uncertainty that arise from sampling and analysis of environmental samples, the following aspects must be taken into consideration. Firstly, it should be emphasized that environmental data are spatially dependent, whereas statistical calculations assume independent samples. Secondly, the single element concentration is determined by a multitude process, whereas most of statistical tests assume that the sample comes from the same distribution. Moreover, environmental data might be imprecise, depending on different times of sampling, specificities of samples, or sample preparations (Reimann and Garrett 2005; Dołęgowska and Migaszewski 2015). According to these aspects, environmental analysis requires a robust approach, more resistant to outlying values that may come from different sources and may disturb a normal distribution. Robust models adopt that data distribution may diverge from the normal shape, so they can be applied when the assumption of normality is not fulfilled (Hoaglin et al. 2000; Erceg-Hurn and Mirosevich 2008; Filzmoser et al. 2009b; Rostron and Ramsey 2012). In this context, the robust analysis of variance known as the RANOVA method used for calculation of sampling uncertainty is readily applied.

The main advantage of this method is accommodation of outlying values that are down-weighted during calculation, so the final results are more reliable. Unfortunately, the RANOVA method cannot be used when the outlying values exceed 10 % of the total results or when these values are to be treated as a feature of the dataset. The presence of more than 10 % of outliers in a dataset may lead to bimodal or multimodal distribution, so the estimation of variance may be invalid (Lee and Ramsey 2001; Ramsey and Ellison 2007). The RANOVA intends that, during calculations, all values that exceed the relation: mean + *c·σ*_*r*_ (where mean is a classical mean and *σ*_*r*_ is a robust standard deviation) are replaced by mean + *c·σ*_*r*_, whereas all values that exceed mean − *c·σ*_*r*_ are replaced by mean − *c·σ*_*r*_. After this step, the mean and *σ*_*r*_ are recalculated. The process is repeated multiple times, until the arithmetic mean stabilizes (converges) at an acceptable level of accuracy. It can be easily calculated using the ROBCOOP4.EXE program, which is based on an iterative approach and is dedicated to geochemical surveys (Rostron and Ramsey 2012).

### Extreme values—how they should be identified?

The preliminary estimation of element distribution in environmental samples (e.g., plants, soils) may be problematic. Samples derived, for example, from derelict metal ore mining areas where element concentrations result from natural and anthropogenic sources need a special attention. Geochemical changes in the environment induced by human activity lead to enrichment in different elements. These also increase the probability of occurrence of outliers in a dataset that makes the results difficult to interpret. Geochemical datasets always contain outliers that can be defined as variables originating from different processes or sources, which belong to a different population (Grünfeld 2005; Reimann and Garrett 2005). Usually, outliers arise from a sample that diverges from other samples. Hence, their presence in a dataset may cause heavy tails in distribution or bimodality (Hampel et al. 1986; Barnett and Lewis 1994; Templ et al. 2008). To avoid this problem, outliers are often removed from the data prior to computing. However, they carry important information about the study area and they should not be ignored, even though their presence disturbs the normal distribution, which is required in a classical analysis of variance. In general, classical models are unsuitable for datasets containing outliers, and the results obtained by these methods can be erroneous (van der Laan and Verdooren 1987).

Identification of outliers is not a trivial task (Reimann and Garrett 2005; Filzmoser et al. 2008), and their amount is a criterion in applying of the RANOVA. The knowledge about statistical distribution of results may be obtained from histograms that belong to the most popular statistical graphics. Unfortunately, the presence of outliers in a dataset makes them commonly useless. As mentioned before, the outliers may be removed or their influence may be reduced through their transformation, but the decision about data transformation and the type of transforming function should be based on the assumed geometry inherent in the data not only in the shape of histogram. However, if any operations on outliers are to be taken, they must be properly identified.

The most popular method used for identification of outliers is mean *± c·σ*, where mean is a classical arithmetic mean, whereas *c* is a factor between 1 and 2 but typically set to 1.5. This method allows us to identify about 2.5 % of the upper and lower extreme values. In this method, the extreme values are defined as values in the tails of statistical distribution. Because both mean and standard deviations are strongly dependent on outlying values, this relation seldom gives an appropriate estimation of threshold. In statistics, these two parameters illustrate the population mean and standard deviations, but sometimes, they may represent the second distribution arising from the presence of outliers in a dataset (Reimann et al. 2005).

The better way to deal with outliers and their impact on the data distribution is to use a method, which does not rely on statistical assumptions and is based on parameters, which are robust against outliers. The use of robust parameters makes that the whole relation does not rely on outlying values. In this context, the more adequate procedure for identification of extreme values from environmental results is a median ± 2·*σ*_*r*_ method. It is a direct analogy to mean *± c·σ*, but the mean is replaced by a median value and a standard deviation by a median absolute deviation (*σ*_*r*_) defined as a median of absolute deviations from a median of all data (Tukey 1977; Rousseeuw et al. 2006). This method allows us to identify extreme values that may originate from superimposed processes (e.g., mining activity), not only from the same source (Reimann et al. 2005).

Another method used for preliminary selection of results and identification of outliers is the boxplot method (Hubert and van der Veeken 2007; Dawson 2011). Like histograms, the boxplots give a lot of information about data distribution. In this method, the dataset is divided into four groups (based on the median value), and subsequently, each group is divided into halves. The 25 % of all results are placed in each group. The lines dividing the groups are called quartiles, and the groups are called quartile groups. The central box collects 50 % of the data. The upper quartile indicates that 75 % of the data are below this quartile, and the lower quartile indicates that 25 % of data are below this quartile. When the boxplot is short, it means that our data are similar to each other, whereas the tall boxplot suggests differentiation within the dataset. In this method, each outlier is a value that lies more than one and a half times the length of the box from its either end (Rousseeuw et al. 2006). According to Reimann et al. (2005), the boxplot and the median ± 2·*σ*_*r*_ methods are more adequate for estimation of extreme values from geochemical surveys. In general, the boxplot gives reliable results when the number of outliers is about 15 %, whereas the median ± 2·*σ*_*r*_ is about 15–25 %.

The last of the described method is the mean *± c·σ*_*r*_, where the mean is a traditional arithmetical mean and *σ*_*r*_ is a robust standard deviation defined as a median of absolute differences between duplicated measurements. This method is used for elimination of outlying values during calculation of uncertainty by the ROBCOOP4.EXE program. Because this is based on the arithmetical mean, it can be successfully used when no outliers exist in a dataset or when they comply with the definition of outliers (do not arise from specificity of sampling area). It can be difficult when geochemical data are taken under consideration (Rostron and Ramsey 2012).

## Fieldworks

**Fig. 1:**
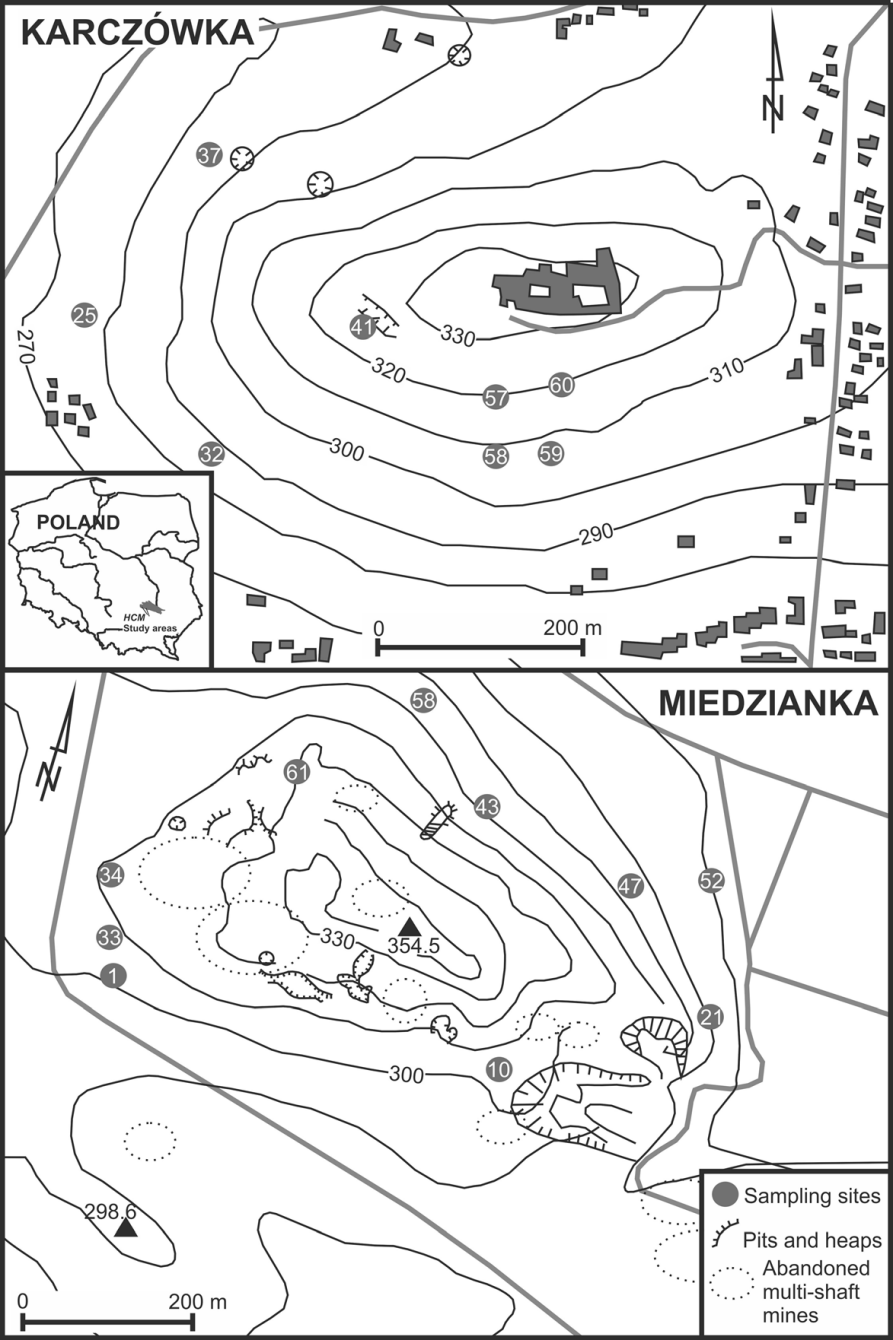
Location of the investigated areas and sampling sites

Soil samples were collected within two remote historic metal ore mining areas: Miedzianka Mount nature reserve (354 m a.s.l.) and Karczówka Mount landscape reserve (335 m a.s.l.) in November of 2012. These sites are located in the southwestern and north-central parts of the Holy Cross Mountains, south-central Poland. Miedzianka Mt. was a significant copper ore mining center until the twentieth century, while Karczówka Mt. was one of the most important lead ore mining centers in the sixteenth to seventeenth century. Eight composite and duplicate samples (each consisted of five to ten increments) from Karczówka and ten samples of the same pattern from Miedzianka were collected within an area of about 1 m^2^ (Fig. 1 of ESM 1). All samples (about 2 kg each) were taken using a systematic random sampling strategy from a depth of about 0.3–0.5 m. The samples were in situ cleaned from alien material, oversized particles (*Ø* > 2 mm), and homogenized. The duplicate samples were collected at a distance of about 1–2 m using the same procedure (Jung and Thornton 1997; Ramsey and Argyraki 1997). All samples were finally transported to the Geochemical Laboratory of the Institute of Chemistry and prepared for further analysis.

## Materials and methods

Soil samples were dried at an ambient temperature and disaggregated to pass a 0.063-mm sieve using a Pulverisette 2 Fritsch grinder and an Analysette 3 Spartan shaker (FRITSCH, Germany). The soil samples (0.5 g each) were digested in a closed microwave system Multiwave 3000 (Anton Paar, Austria) according to the procedure presented in Table 1. After digestion had been completed, solutions were replenished up to 25 mL. The concentrations of As, Cd, Co, Cr, Cu, Mn, Ni, Pb, and Zn were determined using the ICP-MS method (ELAN DRC II, PerkinElmer, USA), and according to the *balance strategy*, each sample was analyzed twice [38]. Instrumental and data acquisition parameters of the ICP-MS instrument are summarized in Table 1. During analysis, two internal standards Rh and Ir and two standard reference materials CRM NIST 2710a (Montana I Soil) and GSS4 (Chinese Academy of Geological Sciences) were applied. The average recovery was in the range of 92–110 %.

**Table 1:** Parameters of digestion process and ICP-MS instrument

Digestion parameter		ICP-MS instrument parameters	
Power	1000 W	Plasma power	1275 W
Time	65 min	Lens voltage	7.50 V
Time of growth	15	Plasma gas flow	15 L min^−1^
Time of real digestion	30	Nebulizer gas flow	1.03 L min^−1^
Time of cooling	20	Sweeps/reading	20
Temperature	220 °C	Readings/replicate	3
Pressure	6 MPa	Replicates	4
p growth rate	0.03 MPa s^−1^	Dwell time	50–150 μs depending on the analyte
Reagents	HCl (6 mL), HNO_3_ (2 mL) MERC		

## Results and discussion

Statistical distribution of results strongly depends on element concentrations. Differences in element contents within sampling area may signify its heterogeneity, which can be a fundamental source of sampling error (Gy 1995; Hildebrandt et al. 2012). Both sampling and analytical uncertainty are dependent on heterogeneity of sampling area, so prior to any statistical estimation of these components, the spatial distribution of elements should be evaluated (Petersen et al. 2005; Bodnar et al. 2013). One of the most popular methods used for identification of homogenous groups of objects within a dataset is a cluster analysis (Templ et al. 2008). This is based on similarities or dissimilarities between data and classifies the obtained results into clusters, which are more practicable and more accessible for interpretation than the original data. In this method, each single cluster depicts a group of homogenous observations (elements) that are similar to one another but are different from elements of other groups (Filzmoser et al. 2009b). To indicate a homogenous group within this study area, the cluster analysis was done with STATSOFT Statistica Software®. At the beginning, the results were normalized with the Box-Cox method and standardized. Subsequently, the analysis was completed using Ward’s method with a square of Euclidian distance as a measure of similarity. The dendrograms were performed at a distance reported as 100·*D*/*D*_max_.

**Fig. 2:**
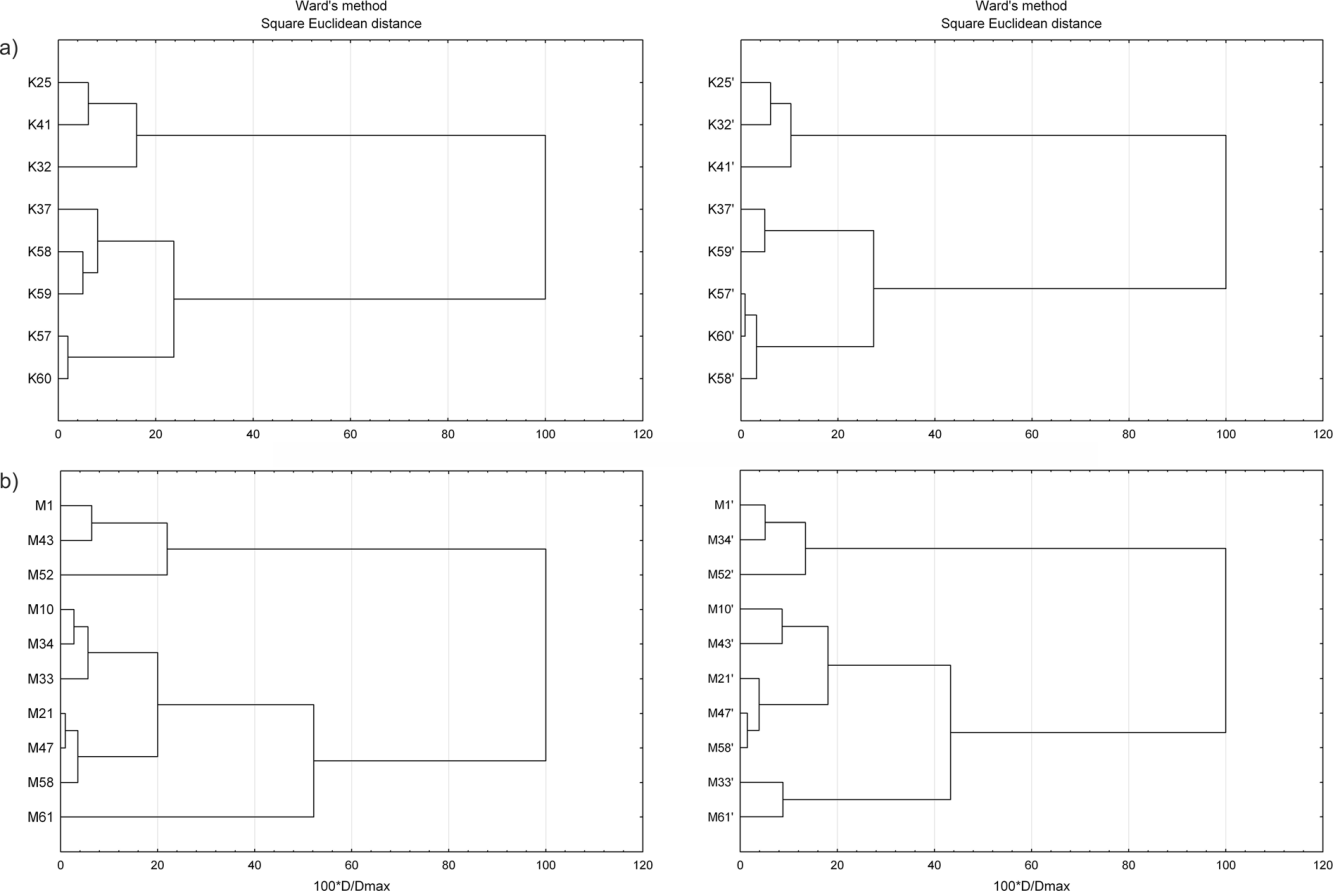
Dendrograms of primary and duplicate sites from **a** Karczówka and **b** Miedzianka

The spatial variability in element concentration was found within the sampling area as well as between primary and duplicate sites selected at the same sampling location. Two identical homogenous groups are observed within hierarchical clustering dendrograms (Fig. 2a) obtained for primary and duplicate sampling sites from Karczówka. The first cluster is formed by sites 25, 32, and 41 whereas the other by sites 37, 57, 58, 59, and 60. The chemical analysis has shown that the highest average concentrations of As, Co, Ni, Zn, Cd, and Pb are noted in soil samples collected from sites forming the first cluster. The Pb levels were even 20 times higher than those noted at sites forming the second cluster. Two main clusters are also recorded in dendrograms obtained for sampling sites from Miedzianka, but the bounding observed between primary and duplicate sites is different (Fig. 2b). The dendrogram of primary samples shows two main groups. The first group is formed by sites 1, 43, and 52 and the other by sites 10, 33, 34, 21, 47, and 58, as opposed to site 61, which forms an independent bundle. It should be stressed that the highest concentrations of all the elements (except for Pb) were recorded at site 61. Two clusters are also displayed in the dendrogram of duplicate samples, but the first cluster is formed by sites 1, 34, and 52 and the other by 10, 21, 33, 43, 47, 58, and 61. Moreover, within the second band, two separate bundles are found; the first is formed by sites 33 and 61 and the other by sites 10, 21, 43, 47, and 58. The coefficient of variance, which is a normalized measure of dispersion of a probability distribution, was also calculated, and it varied from 36 to 140 % for elements from Karczówka and from 40 to 170 % for elements from Miedzianka. This also indicates that the differences within datasets are statistically significant.

**Fig. 3:**
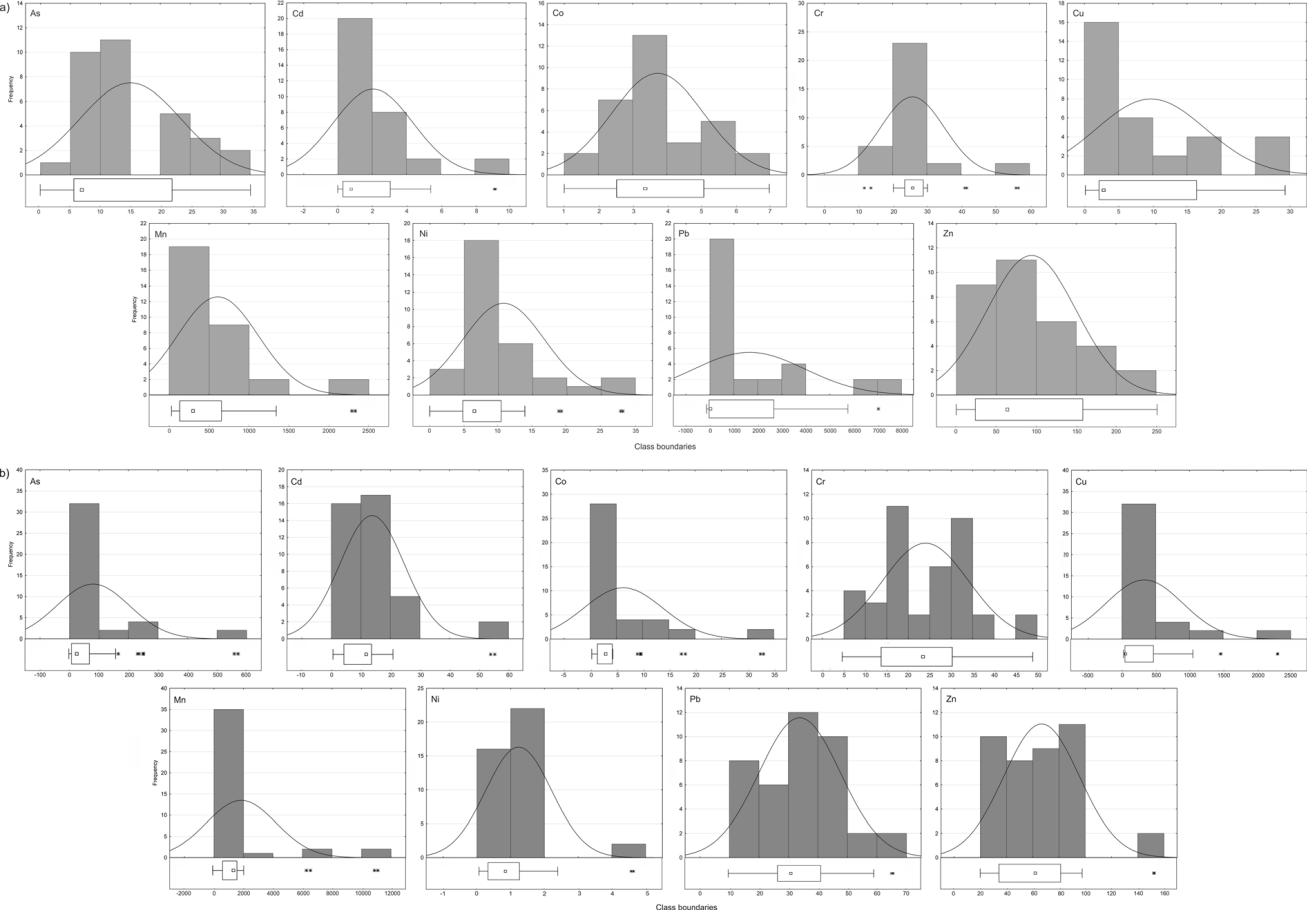
Histograms and boxplots of data distribution from **a** Karczówka and **b** Miedzianka

To check the normal distribution of datasets, the histograms of each element were prepared and *p* values using Shapiro-Wilk test were calculated. The analysis of histograms and *p* values (95 %) shows that results for Co (0.11) from Karczówka and Cr (0.06) and Pb (0.09) from Miedzianka are normally distributed. The sampling uncertainty (*s*_*r*samp_ (%)) calculated with the ANOVA method for Co, Cr, and Pb is 15.6, 20.2, and 38.7 %, respectively. The other elements exhibit a skewed right distribution, and for these elements, outlying values were identified with the following methods: boxplots, mean *± c·σ*, mean ± 1.5·*σ*_*r*_, and median ± 2·*σ*_*r*_. The percentage of detected outliers is different and depends on the method used (as shown in Table 2 of ESM 2). The boxplots find more than 10 % of extreme values only for Cr and Ni from Karczówka and for As and Co from Miedzianka (as shown in Fig. 3a, b of ESM 1). No outlying values in the most datasets examined have been found, but the length of “boxes” may suggest their differentiation. This is also confirmed by the analysis of histograms. The mean *± c·σ* and the median ± 2·*σ*_*r*_ procedures give a completely different outcome. More than 10 % of outliers are identified with the median ± 2·*σ*_*r*_ method for all the elements determined in the soil samples from Karczówka and for As and Cu from Miedzianka, i.e., the elements with the highest coefficient factor (154 and 171 %, respectively). The amount of extreme values, which have been identified using the mean *± c·σ* method, is dependent on the *c* factor. When the *c* factor is 1.5, more than 10 % of extreme values are identified only for As, Cu, Ni, Pb, and Zn from Karczówka. In case that the *c* factor is 2, the extreme values are not observed in any dataset. Using the mean ± 1.5·*σ*_*r*_ method, more than 10 % of outliers in all the examined datasets are identified.

**Table 2:** Percentage of detected outliers identified with four different methods

	As	Cd	Co	Cr	Cu	Mn	Ni	Pb	Zn
Boxplots									
Karczówka	<10 %	<10 %	Normal distribution	>10 %	<10 %	<10 %	>10 %	<10 %	<10 %
Miedzianka	>10 %	<10 %	>10 %	Normal distribution	<10 %	<10 %	<10 %	Normal distribution	<10 %
Mean ± 1.5·*σ*									
Karczówka	>10 %	<10 %	Normal distribution	<10 %	>10 %	<10 %	>10 %	>10 %	>10 %
Miedzianka	<10 %	<10 %	=10 %	Normal distribution	=10 %	=10 %	<10 %	Normal distribution	<10 %
mean ± 2.0·*σ*									
Karczówka	<10 %	<10 %	Normal distribution	<10 %	<10 %	<10 %	<10 %	<10 %	<10 %
Miedzianka	<10 %	<10 %	<10 %	Normal distribution	<10 %	<10 %	<10 %	Normal distribution	<10 %
mean ± *c*·*σ*_*r*_									
Karczówka	>10 %	>10 %	Normal distribution	>10 %	>10 %	>10 %	>10 %	>10 %	>10 %
Miedzianka	>10 %	>10 %	>10 %	Normal distribution	>10 %	>10 %	>10 %	Normal distribution	>10 %
median ± 2·*σ*_*r*_									
Karczówka	>10 %	>10 %	Normal distribution	>10 %	>10 %	>10 %	>10 %	>10 %	>10 %
Miedzianka	>10 %	<10 %	=10 %	Normal distribution	>10 %	=10 %	=10 %	Normal distribution	<10 %

In summary, the boxplot method combined with the histograms gives primary information about data distribution, but this information may be ambiguous (*see* the boxplots of elements from Karczówka, Fig. 3a of ESM 1). The histograms allow us to inspect the data distribution, whereas boxplots show the median and skewness of the distribution and allow for preliminary identification of data outliers. The methods that use an arithmetic mean, which is strongly influenced by the extreme values, may also disturb the correct interpretation of results. Hence, the method that eliminates the direct relation with the arithmetic mean and down-weights the impact of outliers, such as the median ± 2·*σ*_*r*_ method, may be the most suitable in the analysis of geochemical data.

Because the RANOVA method may be applied only when the outlying values do not exceed 10 % of the total results, the sampling uncertainty can be calculated with this method for

- As, Cd, Cu, Mn, Pb, and Zn from Karczówka and Cd, Cu, Mn, Ni, and Zn from Miedzianka—after identification of outlying values with the boxplot method
- Cd, Cr, and Mn from Karczówka and all the determined elements from Miedzianka—after identification of outlying values with the mean *±* 1.5·*σ* method
- All the elements from Karczówka and Miedzianka—after identification of outlying values with the mean ± 2.0·*σ* method
- None of these elements—after identification of outlying values with the mean *±* 1.5·*σ*_*r*_ method
- Cd, Co, Mn, Ni, and Zn from Miedzianka—after identification of outlying values with the median ± 2·*σ*_*r*_ method

**Fig. 4:**
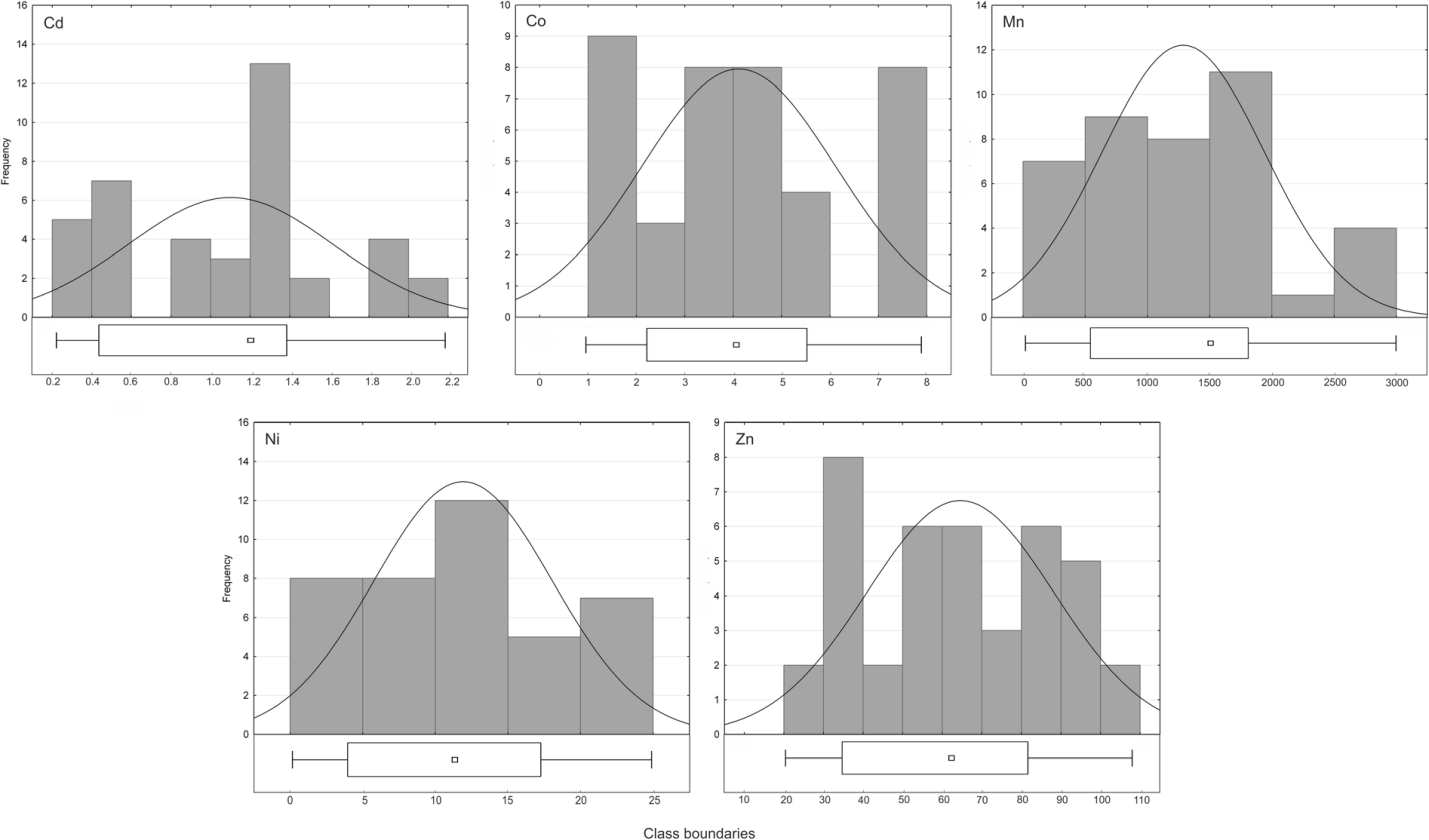
Histograms and boxplots of data distribution after recalculation process

In the RANOVA method described by Rostron and Ramsey (2012), the outlying values are defined as values exceeding the relation mean *± c·σ*_*r*_ (vide subsection Extreme values – how they should be identified?). Because the median ± 2·*σ*_*r*_ technique is more suitable for identifying outliers in geochemical data, we used it by analogy to Rostron and Ramsey (2012) to calculate the sampling uncertainty. During the calculation process, all extreme values lower than median − 2·*σ*_*r*_ were replaced by median − 2·*σ*_*r*_ and all higher than median + 2·*σ*_*r*_ were replaced by median + 2·*σ*_*r*_. After this process was completed, the median (if it changed) and robust standard deviation were recalculated. After each operation, the histograms were made and datasets were tested for normality. The statistical operation was repeated as *p* value (calculated with Shapiro-Wilk test) was constant or when normality was achieved. The sampling uncertainty (*s*_*r*samp_ (%)) calculated with this method for Cd, Co, Mn, Ni, and Zn from Miedzianka was as follows: 28.9 % for Cd, 15.2 % for Co, 12.7 % for Ni, 14.5 % for Mn, and 16.3 % for Zn. The sampling uncertainty calculated with a traditional RANOVA model using the ROBCOOP4.EXE program was 16.7 % for Cd, 9.2 % for Co, 20.5 % for Mn, 17.9 % for Ni, and 16.3 % for Zn. We decided to compare these results and calculate uncertainty with a traditional RANOVA method because the amount of outliers recognized with the mean ± 1.5·*σ* technique was lower than 10 % for the elements examined. The higher sampling uncertainty computed with a modified model compared to a traditional one was obtained for Cd and Co. As for Cd, during a recalculating process, the element distribution approached multimodality (as shown in Fig. 4 of ESM 1).

The calculation process was completed when the *p* value began to decrease. This may suggest that, for this element sampling, uncertainty should be calculated only after data transformation. As for Co, the distribution shape has changed as shown in Fig. 4, and the *p* value has increased from 0.000 to 0.003. It is noteworthy that for Mn, Ni, and Zn, the recalculating process led to achieve normality. The final uncertainty computed with this method compared to the uncertainty computed with a traditional model was lower for Mn and Ni and remained unchanged for Zn. Using the boxplot method, we did not find the outlying values in the datasets modified by recalculation process (Fig. 4 of ESM 1), but the “box length” still pointed to their differentiation.

## Conclusions

The following conclusions can be drawn from the datasets obtained from this study:

1. The spatial distribution of elements in heterogeneous areas is difficult to predict. The outlying values identified in the datasets derived from the analysis of soil samples may not comply with the statistical definition of outliers.
2. The level of sampling uncertainty assessed for soil samples may be high (even above 30 %) and may arise from heterogeneity of the study area, which is the fundamental source of sampling errors.
3. The RANOVA can be successfully used to calculate the uncertainty arising from sampling provided that the outlying values in each dataset are properly identified.
4. Different methods used for identification of outlying values may give completely different outcome. The selection of correct method should always precede complex characterization of a study area and localization of potential sources of elements.
5. The graphical methods, e.g., histograms or boxplots, give preliminary information about the data distribution. The presence of outliers in a dataset commonly makes them useless, so their analysis must be done thoroughly, and they cannot be the sole source of our knowledge about the data distribution.
6. The modified RANOVA method using the median ± 2·*σ*_*r*_ procedure for elimination of outlying values during the calculation process is more suitable for assessing the sampling uncertainty of results derived from geochemical studies. The use of robust parameters makes them independent on outliers which, on the other hand, cannot be eliminated in geochemical studies. It should be noted that they always carry important information about the study area.

## Electronic supplementary material

ESM 1 (PDF 706 kb)

ESM 2 (DOCX 15 kb)
