# Supplementary material for: Heterogeneous areas—identification of outliers and calculation of soil sampling uncertainty using the modified RANOVA method
Source: Environ Monit Assess. 2016 Sep 22;188(10):581. doi: 10.1007/s10661-016-5584-9 (PMC5033995; doi:10.1007/s10661-016-5584-9)
Supplement: Supplementary file 1 — (PDF 706 kb) [file 10661_2016_5584_MOESM1_ESM.pdf]

Environmental Monitoring and Assessment, Springer, Heidelberg, 2016

Electronic supplementary materials for:

Heterogeneous areas – identification of outliers and calculation of soil sampling uncertainty using the modified RANOVA method

Sabina Dołęgowska*[a], Agnieszka Gałuszka[a], Zdzisław M. Migaszewski[a]

[a]Geochemistry and the Environment Division, Institute of Chemistry, Jan Kochanowski University, 15G Świętokrzyska St., 25-406 Kielce, Poland

*Corresponding author: Sabina.Dolegowska@ujk.edu.pl

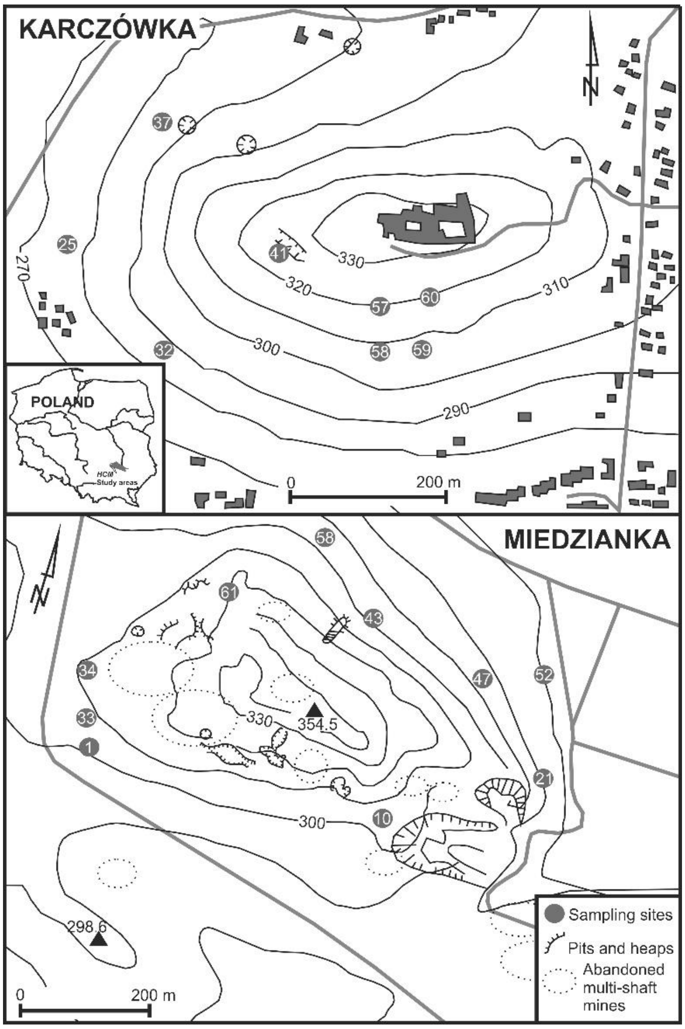

Fig. 1 Location of the investigated areas and sampling sites.

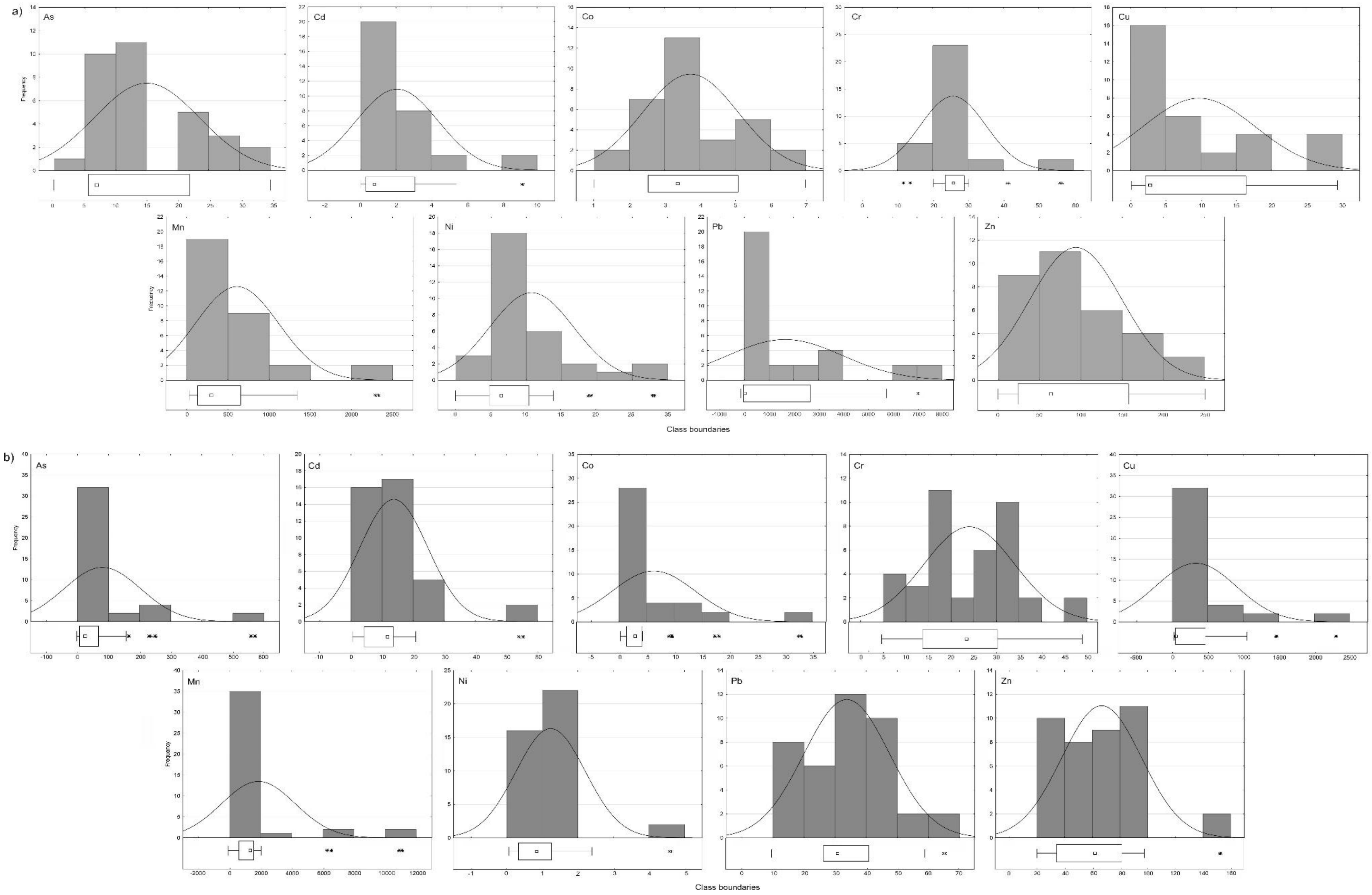

Fig. 3 Histograms and boxplots of data distribution from: a – Karczówka; b – Miedzianka

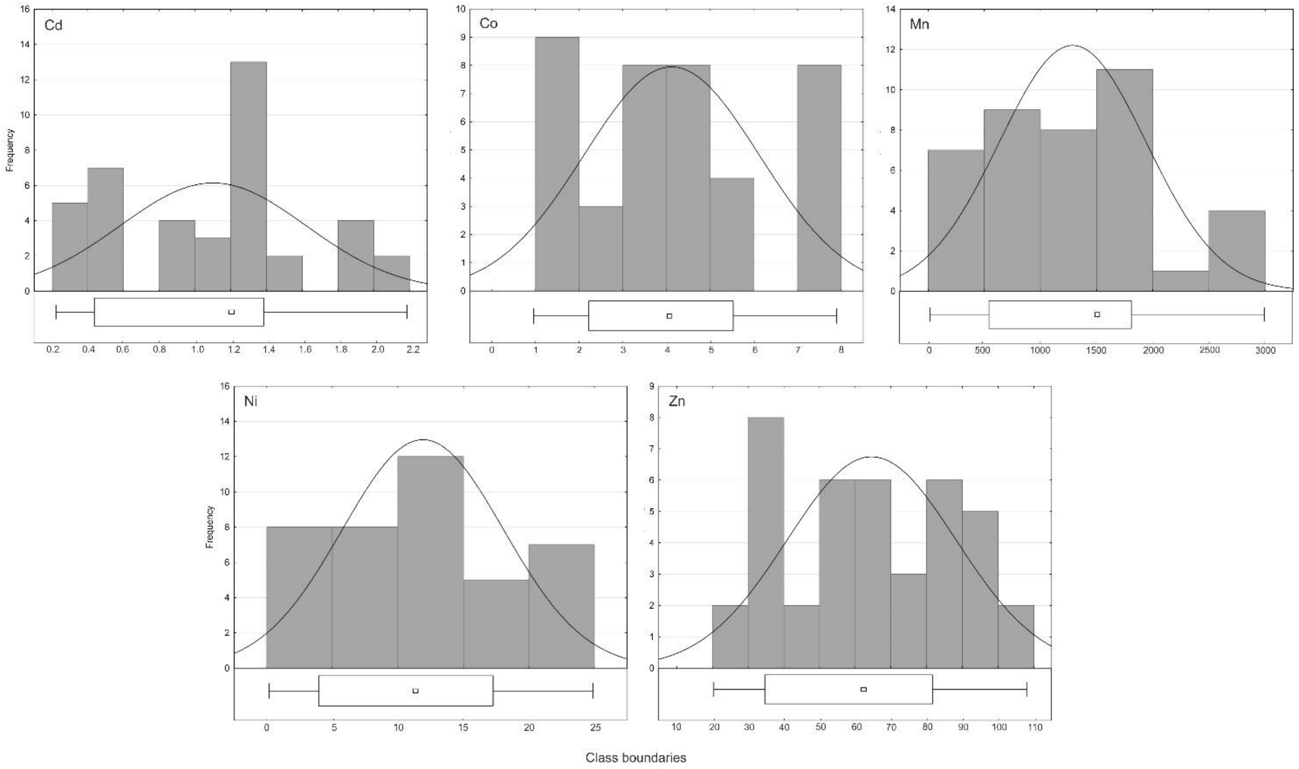

Fig. 4 Histograms and boxplots of data distribution after recalculation process.
