# Supplementary material for: Heterogeneous areas—identification of outliers and calculation of soil sampling uncertainty using the modified RANOVA method
Source: Environ Monit Assess. 2016 Sep 22;188(10):581. doi: 10.1007/s10661-016-5584-9 (PMC5033995; doi:10.1007/s10661-016-5584-9)
Supplement: Supplementary file 2 — (DOCX 15 kb) [file 10661_2016_5584_MOESM2_ESM.docx]

Environmental Monitoring and Assessment, Springer, Heidelberg, 2016

Electronic supplementary materials for:

**Heterogeneous areas – identification of outliers and calculation of soil sampling uncertainty using the modified RANOVA method**

Sabina Dołęgowska*^a^, Agnieszka Gałuszka^a^, Zdzisław M. Migaszewski^a^

^a^Geochemistry and the Environment Division, Institute of Chemistry, Jan Kochanowski University, 15G Świętokrzyska St., 25-406 Kielce, Poland

*Corresponding author: Sabina.Dolegowska@ujk.edu.pl

**Table 2** Percentage of detected outliers identified with four different methods

|  | As | Cd | Co | Cr | Cu | Mn | Ni | Pb | Zn |  |  |
| --- | --- | --- | --- | --- | --- | --- | --- | --- | --- | --- | --- |
| boxplots | | | | | | | | | | |  |
| Karczówka | <10 % | <10 % | Normal distribution | >10 % | <10 % | <10 % | >10 % | <10 % | <10 % |  |  |
| Miedzianka | >10 % | <10 % | >10 % | Normal distribution | <10 % | <10 % | <10 % | Normal distribution | <10 % |  |  |
| mean±1.5·σ | | | | | | | | | | | |
| Karczówka | >10 % | <10 % | Normal distribution | <10 % | >10 % | <10 % | >10 % | >10 % | >10 % |  |  |
| Miedzianka | <10 % | <10 % | =10 % | Normal distribution | =10 % | =10 % | <10 % | Normal distribution | <10 % |  |  |
| mean±2.0·σ | | | | | | | | | | | |
| Karczówka | <10 % | <10 % | Normal distribution | <10 % | <10 % | <10 % | <10 % | <10% | <10 % |  |  |
| Miedzianka | <10 % | <10 % | <10 % | Normal distribution | <10 % | <10 % | <10 % | Normal distribution | <10 % |  |  |
| mean±c·σ_r_ | | | | | | | | | | | |
| Karczówka | >10 % | >10 % | Normal distribution | >10 % | >10 % | >10 % | >10 % | >10% | >10 % |  |  |
| Miedzianka | >10 % | >10 % | >10 % | Normal distribution | >10 % | >10 % | >10 % | Normal distribution | >10 % |  |  |
| median±2·σ_r_ | | | | | | | | | | | |
| Karczówka | >10 % | >10 % | Normal distribution | >10 % | >10 % | >10 % | >10 % | >10% | >10 % |  |  |
| Miedzianka | >10 % | <10 % | =10 % | Normal distribution | >10 % | =10 % | =10 % | Normal distribution | <10 % |  |  |
